# Do Tumor Mechanical Stresses Promote Cancer Immune Escape?

**DOI:** 10.3390/cells11233840

**Published:** 2022-11-30

**Authors:** Killian Onwudiwe, Julian Najera, Saeed Siri, Meenal Datta

**Affiliations:** Department of Aerospace and Mechanical Engineering, University of Notre Dame, Notre Dame, IN 46556, USA

**Keywords:** solid tumors, autophagy, epithelial–mesenchymal transition, immune evasion, fluid shear stress, solid stress, interstitial fluid pressure, immunotherapy

## Abstract

Immune evasion—a well-established cancer hallmark—is a major barrier to immunotherapy efficacy. While the molecular mechanisms and biological consequences underpinning immune evasion are largely known, the role of tissue mechanical stresses in these processes warrants further investigation. The tumor microenvironment (TME) features physical abnormalities (notably, increased fluid and solid pressures applied both inside and outside the TME) that drive cancer mechanopathologies. Strikingly, in response to these mechanical stresses, cancer cells upregulate canonical immune evasion mechanisms, including epithelial–mesenchymal transition (EMT) and autophagy. Consideration and characterization of the origins and consequences of tumor mechanical stresses in the TME may yield novel strategies to combat immunotherapy resistance. In this Perspective, we posit that tumor mechanical stresses—namely fluid shear and solid stresses—induce immune evasion by upregulating EMT and autophagy. In addition to exploring the basis for our hypothesis, we also identify explicit gaps in the field that need to be addressed in order to directly demonstrate the existence and importance of this biophysical relationship. Finally, we propose that reducing or neutralizing fluid shear stress and solid stress-induced cancer immune escape may improve immunotherapy outcomes.

## 1. Introduction

**Figure 1:**
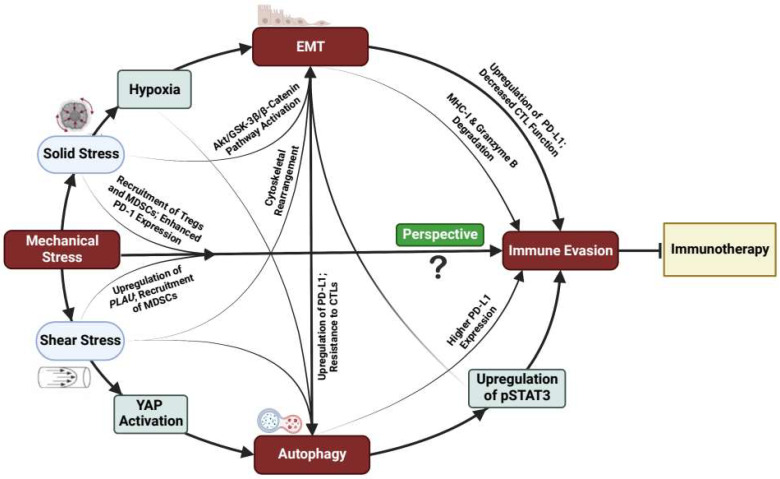
The physical and biological characteristics of tumors and their microenvironment interact to influence immune evasion and immunotherapy outcomes. Mechanical stress (solid and fluid) can exert both direct and indirect effects to promote immune evasive mechanisms. In tumors, solid stress (compressive and tensile) collapses tumor blood vessels, causing hypoxia within the TME, and induces EMT and autophagy within cancer cells via the activation of pathways responsible for mesenchymal transition and invasiveness [6,7,8,9,10,11,12]. The solid stress-induced activation of other pathways (e.g., VEGF) in turn enhances PD-1 expression and the recruitment of Tregs and MDSCs, thus promoting immunosuppression [13,14]. In the presence of fluid components within the TME, and during circulation, cancer cells experience increased fluid shear stress, which can directly enhance and support immune evasion via the recruitment of MDSCs and upregulation of PD-L1 [15,16,17,18,19,20]. Shear stress also induces EMT and autophagy mechanisms by promoting cytoskeletal rearrangement and the formation of autophagosomes [21,22,23,24]. The mechanical induction of EMT and autophagy by solid and shear stresses may mediate cancer immune escape via variable mechanisms that have yet to be directly tested, though indirect evidence exists that suggests that some of the following phenomena may be involved. EMT inhibits the CTL-mediated killing of malignant cells and promotes immune evasion due to increased resistance to CTL-activated death receptor pathways and the enhanced expression of PD-L1 [25,26,27]. Autophagy also supports immune evasion via the degradation of MHC-I and granzyme B, and the upregulation of pSTAT3 [28,29,30]. Interplay between EMT and autophagy influences immune evasion, although this remains controversial [31,32]. We postulate that this collective contribution of aberrant tumor mechanical stresses on immunological responses may inhibit immunotherapy efficacy.

Several studies have shown tremendous progress in the development of different therapies to mitigate metastasis and cancer cell growth. Immunotherapies such as adoptive cell transfer (ACT) and immune checkpoint blockade (ICB) have had a tremendous impact on the treatment of various cancers [1]. However, resistance to immunotherapy remains a major challenge in the clinic [2,3]. This is primarily due to the ability of cancer cells to evade the anti-tumoral immune response [3]. Therefore, understanding and targeting mechanisms of immune evasion may greatly enhance the immunotherapeutic efficacy in patients. Cellular processes that promote cancer immune escape include autophagy and epithelial–mesenchymal transition (EMT) [4,5]. Interestingly, the induction of autophagy and EMT has been observed in cancer cells under mechanical stress—specifically fluid shear and solid stresses (Figure 1). However, to our knowledge, fluid shear and solid stress have not yet been shown to directly mediate immune evasion via autophagy and EMT. We define these mechanical stresses and discuss their linkages to immune evasion in the following sections. Lines of investigation into these mechanopathological interactions will facilitate further understanding of cancer cell adaptability and the survival response and may serve as a promising avenue in overcoming resistance to immunotherapy.

## 2. Roles of EMT and Autophagy in Immune Evasion

The term “immune evasion” encompasses numerous mechanisms by which cancer cells evade anti-tumor immune responses and destructive immunity; thus, it poses a major barrier to effective cancer immunotherapy [1,3]. Here, we briefly summarize two such mechanisms that have been thoroughly described elsewhere: EMT and autophagy [33,34,35,36,37,38]. EMT is a cellular process characterized by a decrease in epithelial markers and the acquisition of mesenchymal traits [39]. Unlike epithelial cells, mesenchymal cells often have increased invasive potential, are more resistant to apoptosis, are more motile, and produce more extracellular matrix (ECM) [40]. Cancer cells can even exist in hybrid EMT states, wherein they possess both epithelial and mesenchymal markers, and may be more prone to tumorigenesis and metastasis [41,42,43]. The transition to a mesenchymal state also dampens the anti-tumoral immune response, further supporting cancer cell survival and progression. For example, EMT is associated with the reduced cytotoxic T cell (CTL)-mediated killing of malignant cells due to their increased resistance to CTL-activated death receptor pathways [25] and the enhanced expression of the immune checkpoint molecule PD-L1 [26,27]. Likewise, TGF-β—a cytokine known to induce EMT [44]—is also often upregulated in cancer cells with mesenchymal features [5]. TGF-β is particularly notorious for its ability to inhibit the proliferation, function, and/or differentiation of anti-tumoral CD8^+^ T cells, type 1 and 2 helper T (T_h1_ and T_h2_) cells, B cells, and NK cells, as well as the activation and maturation of dendritic cells (DCs) [45,46,47]. TGF-β also enhances the pro-tumoral immune response by promoting the function of regulatory T cells (Tregs) (which also secrete TGF-β), facilitating the conversion of normal fibroblasts into cancer-associated fibroblasts (CAFs), and polarizing macrophages and neutrophils to an M2/N2 (i.e., tumor-promoting) phenotype [47,48,49]. Therefore, TGF-β is involved in a vicious cycle that continuously promotes the suppression of anti-tumor immune activity. Collectively, EMT-induced immune evasion attenuates the efficacy of immunotherapeutic treatment. Indeed, EMT is currently under investigation as a predictive biomarker for immunotherapy response [5].

Similar to EMT, autophagy facilitates tumor cell escape from immunity and mediates immunotherapeutic resistance [4,50]. Autophagy is a catabolic process that involves the degradation of intracellular components (proteins, lipids, and mitochondria) to promote cell survival under environmental stresses (e.g., nutrient deficiency). In cancer, it plays a dual role in modulating the immune response [4]. On the one hand, autophagy promotes the degradation of MHC-I [29]—a cell surface protein required for T cells to recognize tumor cells via antigen presentation [51]—and granzyme B, a serine protease used by CTLs and natural killer (NK) cells to initiate apoptosis in cancer cells [28]. Moreover, autophagy has been observed to foster an immunosuppressive environment by promoting pSTAT3 accumulation [30]. On the other hand, the autophagic degradation of PD-1/PD-L1 immune checkpoint molecules can also render cancer cells more susceptible to T cell-mediated killing [52,53].

Interestingly, crosstalk between EMT and autophagy has been observed. However, autophagy can serve as both a positive and negative regulator of EMT depending on the cell type and state of progression through the metastatic process [31]. In breast cancer cells, for example, the acquisition of a mesenchymal phenotype was observed to promote resistance to CTL-mediated killing via autophagy activation [54]. Likewise, through the activation of EMT, autophagy has been linked to enhancing the invasive potential of hepatocellular carcinoma (HCC) cells [55]. On the contrary, high expression of death effector domain-containing DNA-binding protein (DEDD)—a protein responsible for mediating apoptosis—can reverse EMT by inducing the selective autophagic degradation of key EMT inducers [56]. Inhibition of autophagy has also been shown to promote EMT, metastasis, and glycolysis in gastric cancer [57]. While the complex interplay between these two processes remains controversial [31,32], EMT and autophagy play an important role in mediating cancer immune escape and, consequently, resistance to immunotherapy. Recently, mechanical stresses, namely fluid shear and solid stresses, have been revealed as important players in activating EMT and autophagy in cancer cells [6,12,21,58,59]. However, the direct relationship between tumor-derived mechanical stresses and immune evasion facilitated by EMT and/or autophagy has not been sufficiently explored in the field. We further propose that targeting these stresses may improve the clinical response to immunotherapy by overcoming EMT- and/or autophagy-mediated immune evasion.

## 3. Fluid Shear and Solid Stresses Induce Autophagy

Fluid and solid mechanical stress (herein referred to as shear stress and solid stress, respectively) have been linked to tumorigenesis, invasion [60,61], and—as discussed in this section—the induction of autophagy. There are two sources of shear stress that a cancer cell may experience throughout its lifetime: (1) shear stress generated by the interstitial fluid flow in the TME, and (2) shear stress within the bloodstream during intravasation and circulation [62]. The latter is also referred to as hemodynamic shear stress, and cancer cells that have entered circulation are termed circulating tumor cells (CTCs) [62]. In comparison to the stress generated by interstitial flow (0.1–1 dyn/cm^2^), cells typically experience higher levels of shear stress when they are in the bloodstream (1–30 dyn/cm^2^) [63]. Subjecting cells to shear stress allows for the mechanical inference of cell states as, in most cases, cancer cells are more compliant (i.e., exhibit elastic deformation) than non-malignant cells under shear stress [64,65]. Autophagy is induced in circulation in some cancer cells as a mechanical stress-induced survival mechanism. For example, lipid rafts—cholesterol- and sphingolipid-rich microdomains of the plasma membrane [66]—serve as mechanotransducers that promote protective autophagy in HeLa cells under exposure to physiological levels (20 dyn/cm^2^) of shear stress [22]. Even lower levels of shear stress (~1–2 dyn/cm^2^) have also been observed to induce autophagic flux in cancer cells. Applying 1–1.4 dyn/cm^2^ of shear stress activates integrin/cytoskeletal pathways in HCC cells, which leads to cytoskeletal rearrangement and, consequently, autophagosome formation [23,24]. While shear stress-induced autophagy has been shown to enhance cell migration and invasion, its relationship with the immune response has not been investigated. Thus, studies directly linking shear stress and autophagy-mediated immune evasion remain to be performed.

As with shear stress, solid stress has been implicated in promoting autophagy. This mechanical stress arises within the confines of a tumor and its surrounding host tissue as the density of cellular (e.g., CAFs, cancer cells) and ECM (e.g., collagen) components increases during tumor progression [67,68,69,70,71]. Solid stress is also partly governed by the stiffening of the ECM due to increased resistance to tumor expansion [60]. Direct in vivo measurements of solid stress remain a challenge. Nonetheless, some studies have shown that this stress can range from 0.21 kPa to as high as 19.0 kPa in human tumors, wherein levels are higher in cancers that are more desmoplastic (e.g., pancreatic cancer) [69,72]. A consequence of solid stress is that blood and lymphatic vessels become compressed, thereby limiting the transport of oxygen and nutrients and increasing the interstitial fluid pressure [72,73]. Importantly, reduced oxygen transport induces hypoxia, which is known to activate autophagy in cells and has been linked to promoting cancer cell survival [6,7,8]. Solid stress may also directly induce autophagy. For example, it has been shown that compressing the cerebral cortex using a cranial compression window—an in vivo device used to recapitulate the effects of brain tumor-derived solid stress—upregulates degenerative autophagy in the compressed neurons [74]. While neurons are non-malignant, these results suggest that compressive solid stress may have the capacity to directly induce autophagy in malignant cells. Various in vitro explorations support this notion. One study demonstrated that applying 0.773 kPa of compressive stress to encapsulated HeLa cells induces autophagy and, as a result, enhances their invasive potential by promoting MMP-2 secretion and the turnover of paxillin, a focal adhesion protein [75]. Interestingly, this process involves lipid raft-mediated mechanotransduction, similar to the shear stress study described earlier [75]. Moreover, human mammary carcinoma cells subjected to constant compression at 0.25 kPa for 30 min form and accumulate autophagosomes [76]. However, after 90 min, the number of autophagosomes returns to basal levels, and cells adapt to the new mechanical environment by remodeling their cytoskeletal structure [76]. Although compression-mediated autophagy is a transient response in vitro, it does raise the question of whether this type of response is sustained for longer periods in vivo due to continuous tumor growth (i.e., chronic and increasing compression). Likewise, as with shear stress, direct connections between compression-mediated autophagy and immune evasion remain to be performed.

## 4. Fluid Shear and Solid Stresses Promote EMT Activation

Tumor mechanical stresses can also enhance the invasive and metastatic potential of cancer cells via the induction of EMT. For example, it has been shown that when exposed to 1.4 dyn/cm^2^ of shear stress, Hep-2 cells adopt a mesenchymal-like phenotype that is reversed upon stress removal. Visually, this is indicated by the morphological transition from polygon cell shapes to an elongated spindle with well-organized F-actin and abundant lamellipodia/filopodia [21]. This is associated with the downregulation of E-cadherin, upregulation of N-cadherin, and translocation of β-catenin to the nucleus—events that are characteristic of the EMT process. These changes culminate in an increase in cell migration ability [77,78]. Shear stress has also been shown to activate EMT and, as a result, enhance metastasis via activation of the YAP pathway—a mechanosensory pathway that has been previously linked to promoting EMT [79,80]. Additionally, shear stress can trigger EMT in CTCs to promote their survival in circulation. For example, breast CTCs that do not undergo apoptosis display mesenchymal features driven by shear stress-activated JNK signaling and, consequently, the suppression of pro-apoptosis gene p53 upregulated modulator of metastasis (PUMA) [59]. High expression of the EMT genes Bcl-2 and JUN (a downstream target of JNK) positively correlate with poor patient survival [59]. Thus, it is of relevance to determine if circulating immune cells play a role in this process.

Solid stress can also induce EMT in cancer cells, both directly and indirectly. As with autophagy, solid stress-induced hypoxia may activate key transcriptional regulators of EMT. Activation of hypoxia-inducible factor-1 subunit alpha (HIF-1α) under low-oxygen conditions promotes EMT through the regulation of TWIST, an important transcriptional regulator of EMT [9,10]. In HCC, the hypoxia-induced upregulation of β-catenin not only promotes enhanced invasion and metastatic potential, but is also associated with poor patient prognosis [11]. In combination with interleukin 6 (IL-6) secreted by CD4+ T cells, solid stress has also been shown to mediate EMT activation in clear-cell renal cell carcinoma (ccRCC) cells via the Akt/GSK-3β/β-catenin signaling pathway [12]. As with HCC, this correlates with enhanced tumor progression and, by using β-catenin as a prognostic predictor, poor survival in ccRCC patients [12]. As seen with shear stress, cellular crowding, geometric changes, and intracellular forces at cell junctions activate YAP mechanotransduction pathways [81,82]. Overall, although shear and solid stresses are important mediators of EMT, it remains unclear how the immune response, or lack thereof, contributes to this enhanced cancer cell survival due to a lack of direct mechanistic studies.

## 5. Potential Implications of Tumor Mechanical Stress-Induced Immune Evasion on Immunotherapy Outcomes

While the introduction of immunotherapy has revolutionized the treatment landscape of many cancers over the past couple of decades, it continues to face many failures in the clinic [1,2,3]. These failures can be largely attributed to the ability of malignant cells to avoid T cell-mediated cytotoxicity [3]. Therefore, targeting the origins of such immune evasion mechanisms is necessary to improve the patient response in the clinic.

Recent studies have suggested that mechanical abnormalities may serve as major mediators for a poor immunotherapeutic response; thus, they may represent novel targets for augmenting immunotherapeutic efficacy. Malignant cells have been observed to be softer than their non-malignant counterparts due to elevated levels of plasma membrane cholesterol [83]. Stiffening the cortical structure of cancer cells via cholesterol depletion enhances ACT efficacy in mice by promoting the ability of cytotoxic T lymphocytes (CTLs) to kill their targets. Thus, softening of the cortical structure has been identified as a “mechanical immune checkpoint” that can be used by cancer cells to evade immune surveillance [83]. Similarly, reducing solid stress improves the response to ICB therapy and survival in ICB-resistant murine metastatic breast cancer models [84]. Here, TME-activated angiotensin receptor blockers (acid-sensitive nanoparticle formulations) inhibit the activity of CAFs and, consequently, reduce solid stress. This approach promotes an immunostimulatory TME by enhancing T cell function and decreasing immunosuppression, all of which contribute to overcoming resistance to immunotherapy [84]. FAK inhibitors can also decrease solid stress by promoting matrix consumption and have been proven to enhance the response to anti-PD1 immunotherapy in the KPC (Kras, p53, and Cre) mouse model [85,86]. Likewise, TGF-β blockade may also (i) lower solid stress via matrix modulation [87,88,89]; and (ii) when combined with anti-PD-L1 antibodies, it may promote the infiltration of CTLs into the tumor [85]. The confined migration of cancer cells has also been shown to promote resistance to anoikis and NK cell-mediated immune surveillance, although the translational consequences of this have not yet been evaluated [90]. Beyond mechanical stresses, mechanical properties such as stiffness and viscoelasticity can also promote tumor immune escape, as reviewed by M. Wang and colleagues, rationalizing further investigation [91]. However, the direct induction of immune evasion—involving any of the immune cells discussed here—by mechanical stresses has not been casually demonstrated (e.g., via in vivo force application [92]) and the potential relevance to the immunotherapeutic response has not been studied.

There are a limited number of ongoing or planned clinical trials evaluating the combination of mechanical-targeting agents and immunotherapies. This includes the combination of losartan (an anti-fibrotic angiotensin inhibitor) with nivolumab (an anti-PD-1) (NCT03563248), and the anti-fibrotic drug pirfenidone with atezolizumab (an anti-PD-L1) (NCT04467723). The FAK inhibitor defactinib has also been tested in combination with avelumab (an anti-PD-L1) (NCT02943317) and pembrolizumab (an anti-PD-1) (NCT02758587), although the former trial was terminated upon completion of the escalation phase, and the recruitment status of the latter is currently unknown. The paucity of trials exploiting mechanical abnormalities to improve the immunotherapeutic response is clear. Combining existing drugs, such as the YAP inhibitor verteporfin, with other immunotherapies may provide promising avenues for identifying efficacious treatment regimens [93]. Moreover, it is also necessary to investigate the extent to which tumor mechanical stresses confer resistance to immunotherapy via the induction of autophagy- and EMT-mediated immune evasion. To date, there are no causal studies that have directly evaluated this connection. However, the increasing evidence linking solid and shear stresses to these cellular processes suggests that this is a route worth exploring in the journey to enhance immunotherapeutic efficacy. It is important, however, to consider when it would be most practical to target and overcome the effects of these stresses to achieve an optimal response (e.g., neo-adjuvant vs. adjuvant therapies).

Here, we have presented a case for directly evaluating the connection between fluid shear and solid stress-induced autophagy and EMT and immune evasion. However, there are many individual studies that, when considered together, suggest other mechanisms by which responses to these stresses can help cancer cells to avoid immune detection (Figure 1). For example, MDA-MB-231 and BT-474 breast cancer cells, but not MCF-7 and SK-BR-3 breast cancer cells, overexpress VEGF-A due to compression-induced microRNA-9 (miR-9) downregulation [13]. VEGF-A enhances PD-1 expression in VEGFR^+^CD8^+^ T cells, thus demonstrating its ability to dampen the anti-tumor immune response [14]. The fact that mechanical compression promotes miR-9 downregulation in one set of cells, but not the other, highlights the heterogeneity of immune evasion mechanisms between different cell types, and therefore indicates the need to perform direct mechanistic studies. Additionally, the application of shear stress has been shown to upregulate *PLAU* in breast cancer cells [15] and activate YAP in breast and prostate cancer cells [17,18], both of which have been tied to fostering an immunosuppressive environment. Specifically, tumors that express urokinase plasminogen activator (uPA)—the transcriptional product of *PLAU*—recruit uPA receptor positive (uPAR^+^) myeloid-derived suppressor cells (MDSCs) [16], which are immature myeloid cells known to suppress immune activity [94]. Moreover, along with promoting cancer immune escape via PD-L1 upregulation [19,20], YAP has also been implicated in facilitating the recruitment of immunosuppressive MDSCs and regulatory T cells (Tregs) [95]. In general, these series of observations strongly imply a link between tumor mechanical stresses and immune evasion. Overall, few therapeutic strategies targeting solid and shear stresses to improve the response to immunotherapy exist. Therefore, it is imperative that connections such as those that have been presented here be directly investigated to establish novel mechanical vulnerabilities that, when targeted, can boost immunotherapy efficacy.

## 6. Conclusions

Taken together, these studies underscore the importance of evaluating the immunological consequences of mechanically stressed cancer cells to understand and improve the patient response to immunotherapy. Targeting tumor mechanical stresses may be a viable option for augmenting immunotherapeutic efficacy. However, current lines of inquiry have only scratched the surface, as the direct links between tumor mechanical stresses, cancer cell integrity, and immune evasion remain to be fully elucidated. Thus, further mechanistic studies are needed to reveal targetable interactions that may be translated to the bedside.
